# Organization factors influencing quality of work life among seniors' care workers with severe low back pain

**DOI:** 10.1002/1348-9585.12378

**Published:** 2023-01-04

**Authors:** Kazuyuki Iwakiri, Midori Sotoyama, Masaya Takahashi, Xinxin Liu

**Affiliations:** ^1^ National Institute of Occupational Safety and Health Kawasaki Japan

**Keywords:** care worker, job satisfaction, low back pain, quality of work life

## Abstract

**Objectives:**

The prevalence of work‐related low back pain (LBP) is high among care workers and can negatively affect quality of work life (QWL). To improve workplace satisfaction, this study aimed to identify factors influencing QWL among seniors' care workers with severe LBP.

**Methods:**

A questionnaire survey including items on demographics, qualifications, basic job responsibilities, job stressors, LBP severity, QWL, and job satisfaction was conducted in 2018. In total, 1000 senior care facilities were selected via random sampling and eight care workers per institution were asked to complete the survey. Multiple logistic regression analysis was used to identify independent factors influencing QWL of care workers with and without severe LBP.

**Results:**

Data from 1247 care workers with severe LBP and 2009 with nonsevere LBP were included in the analysis. Overall QWL was lower in the severe LBP group than in the nonsevere LBP group. In both groups, human relationships, workplace support, discretionary responsibility level, and working hours or time off were identified as common factors influencing QWL. In the severe LBP group, the salary was also a significant influence on QWL, while in the nonsevere LBP group, the number of workers, promotion or official position, and caregiving technique were identified as significant QWL factors.

**Conclusions:**

The QWL of care workers with severe LBP was strongly influenced by salary. Since care workers suffering from severe LBP are working for a salary while enduring the pain and do not have found a worth doing, they need to prevent LBP and get job satisfaction and self‐progress.

## 1 INTRODUCTION

In Japan, the qualifications and services provided by care workers are distinct from those of nurses. In general, the support provided by care workers is focused on helping patients with activities of daily living. Approximately 50% of care workers in Japan are licensed by the government,^1^ although national qualifications are not always required for caregiving jobs. Many seniors' care facilities are experiencing a shortage of care workers due to attrition^1^ and the increased caseload incurred by rapid population aging.^2^ In fact, the government has estimated that it will be necessary to secure an additional 320 000 care workers by 2025 and 690 000 by 2040.^2^

To attract and retain personnel, a care facility must provide a safe, fair, flexible, and rewarding work environment for care workers to maintain quality of work life (QWL). The concept of QWL is similar to overall job satisfaction. When QWL is stable, job performance and retention increase.^3^ Organizational factors influencing QWL among care workers include salary, human relationships with superiors and colleagues, and the desire to grow through work.^4^ Moreover, high QWL, overall job satisfaction, and low staff turnover require workplace support from superiors,^5^, ^6^, ^7^ opportunities for promotion or official positions,^5^ education and training,^8^, ^9^, ^10^ high discretionary responsibility level,^11^, ^12^, ^13^ steady but flexible working hours,^14^ and a sufficient number of coworkers to handle the workload.^12^, ^15^

Low back pain (LBP) is a serious and pervasive occupational safety and health hazard, and LBP prevalence is high among care facility staff.^16^, ^17^, ^18^, ^19^, ^20^ In Japan, the number of care workers with LBP is increasing in parallel with workforce size,^21^ which as mentioned must expand to meet the needs of an aging population.^2^ Care workers with severe LBP are limited in task abilities. Hence, severe LBP can affect both the care received and QWL. Moreover, the factors influencing QWL may differ from those influencing QWL among care workers without severe LBP. It is critical to identify factors influencing the QWL of care workers with severe LBP to improve patient care, QWL, and staff retention.

Therefore, this study aimed to clarify the relationship between QWL and job satisfaction regarding each task and identify factors influencing QWL among seniors' care workers with severe LBP.

## 2 METHODS

### 2.1 Research design

This cross‐sectional study was conducted in seniors' care facilities across Japan. At the time of study initiation in June 2018, 6940 seniors' care facilities, termed special nursing homes for the aged, were registered in the Ministry of Health, Labor, and Welfare Publication System of Long‐Term Care Service Information directory, and 262 111 individuals were registered as care workers. Among these facilities, 1000 were selected via random sampling (sampling rate of 14.4%), and eight care workers differing in sex, age, and years of experience were selected by the administrator per facility (for a sampling rate of 8000 in 262 111 or 3.1%). An anonymous self‐administered questionnaire was developed and distributed to the selected care workers.

### 2.2 Questionnaire

The questionnaire gathered information on basic demographic and job characteristics, job stressors, LBP severity during the past year, QWL, and job satisfaction regarding each required task.

#### 2.2.1 Basic information

Basic demographic and job characteristics included sex, age, body mass index, smoking status, official qualifications, total years of experience, work time, work shifts, and the total number of working hours per week.

#### 2.2.2 Job stressor

Questions on job stressors were developed based on the job demands, job control, and worksite social support items of the Brief Job Stress Questionnaire.^22^, ^23^ Job demand questions included “I have an extremely large amount of work to do,” “I can't complete work in the required time,” and “I have to work as hard as I can.” Job control questions included “I can work at my own pace,” “I can choose how and in what order to do my work,” and “I can express my opinions on workplace policy.” Responses were measured using the following 4‐point scale: “Very much so” (1 point), “Moderately so” (2 points), “Somewhat” (3 points), and “Not at all” (4 points). Worksite social support questions included “How freely can you talk with the following people?” “How reliable are the following people when you are troubled?” and “How well will the following people listen to you when you ask for advice on personal matters?” The people were the respondent's superiors and coworkers. Again, responses were measured using the following 4‐points scale: “Extremely” (1), “Very much” (2), “Somewhat” (3), and “Not at all” (4). Total scores for job demands and job control responses ranged from 3 (low stress) to 12 (high stress), while total scores for the 6 worksite social support responses ranged from 6 (low stress) to 24 (high stress).

#### 2.2.3 LBP

LBP severity was divided into four grades based on the scheme devised by Von Korff et al.^24^: grade 0, no LBP; grade 1, LBP not interfering with work; grade 2, LBP interfering with work; grade 3, LBP interfering with work and leading to sick leave. Of these, grades 0 and 1 were defined as “nonsevere LBP,” whereas grades 2 and 3 were defined as “severe LBP.”

#### 2.2.4 QWL

Quality of work life was measured using the short‐form scale for QWL among care workers (QWL short‐form scale),^25^ which includes items on “motivation for work,” “volition to continue working,” “satisfaction with work,” and “accomplishment of work.” All questions were scored on a 5‐point scale with the following responses: “very low or weak” (1), “low or weak” (2), “no opinion” (3), “high or strong” (4), and “very high or strong” (5), and the total score (4–20 points) was used to evaluate QWL. The total score was significantly correlated with the scale of QWL for staff in nursing facilities for the elderly,^4^ the work engagement scale in Japanese,^26^ and the Japanese burnout scale.^27^ Therefore, this is a valid and reliable indicator of QWL among care workers. A low score indicates a low QWL, a high score indicates a high QWL, and a middle score indicates neither. In the present study, a total score of 16 points (4 points × 4 items) or more was defined as “high QWL” while a total score of less than 16 points was defined as “low QWL.”

#### 2.2.5 Job satisfaction for each task

Task‐specific job satisfaction was developed and evaluated using items based on previous studies and our interview research. Survey items addressed salary,^4^, ^8^, ^9^, ^10^, ^14^, ^28^ human relationships with superiors and colleagues,^4^, ^8^, ^10^, ^11^, ^14^, ^29^ support from the workplace in case of trouble,^5^, ^6^, ^7^ promotion or official position,^5^ care education and training,^8^, ^9^, ^10^ discretionary responsibility level,^11^, ^12^, ^13^ working hours or time off,^14^ and number of workers.^12^, ^15^ In addition, based on the results of our interviews of 12 care workers in May 2018 to explore the QWL improvement factor (unpublished), we included questions addressing caregiving techniques and personal events. Responses were measured using a 5‐point scale with the following satisfaction levels: “very dissatisfied” (1), “dissatisfied” (2), “no opinion” (3), “satisfied” (4), and “very satisfied” (5). The responses “very dissatisfied”, “dissatisfied,” and “no opinion” were defined as “dissatisfaction” whereas “satisfied” and “very satisfied” were defined as “satisfaction” for analysis.

### 2.3 Survey procedure

All questionnaires were sent to the administrators of selected seniors' care facilities by mail starting in October 2018, and administrators were instructed to distribute the questionnaire to the eight care workers randomly chosen by the administrator. The completed questionnaires were collected from each individual by mail starting in December 2018.

The care workers were fully informed of the study plan and assured that personal information provided in writing was protected. Written informed consent was then obtained from each participant. This study conformed to the principles of the Declaration of Helsinki and was approved by the ethics board of the National Institute of Occupational Safety and Health of Japan (registration ID: H3002).

### 2.4 Data analysis

Questionnaires missing information on sex, age, and severity of LBP were excluded from the analysis. Continuous variables were compared between severe LBP and nonsevere LBP groups by unpaired *t*‐test, while dichotomous variables were compared between groups by *χ* ^2^ test. Within each LBP group, the associations between QWL level (low or high) and job satisfaction parameters (dissatisfaction or satisfaction) were analyzed by multiple logistic regression using the forced entry method. Odds ratios (ORs) and 95% confidence intervals (95% CIs) were calculated for all parameters. The model included sex (male or female), age group (<30, 30–39, 40–49, or ≥50 years), total weekly working hours (<35, 35–39, 40–44, or ≥45 h), job demand score (3–12 points), job control score (3–12 points), and worksite social support score (6–24 points) as independent variables. The independent variables had a variance inflation factor of below 1.5. All statistical analyses were conducted using the Statistical Package for the Social Sciences (IBM SPSS version 27) and *p* ≤ 0.05 was considered significant for all tests.

## 3 RESULTS

### 3.1 Target of analysis

Completed questionnaires were collected from 3565 care workers (response rate of 44.6%), of which 1247 (431 males and 816 females) reported severe LBP and 2009 (798 males and 1211 females) reported nonsevere LBP. The responses of 87 care workers were excluded due to missing sex and age, while data from 222 care workers were excluded due to missing LBP severity levels.

### 3.2 Basic information about care workers

The severe and nonsevere LBP groups showed statistically significant differences for multiple items related to baseline demographic and basic job characteristics (Table 1), but the differences were small. In summary, the severe LBP group included slightly more females, slightly younger individuals, slightly more smokers, and slightly more certified care workers than the nonsevere LBP group. A higher proportion of the severe LBP group had worked for fewer than 10 years, were more engaged in shift work, and worked more than 40 hours/week than was the case for the nonsevere LBP group.

**TABLE 1:** Basic demographic and job characteristics of participating care workers.

(% or mean ± SD)	Severe LBP (*n* = 1247)	Nonsevere LBP (*n* = 2009)	*p* ^a^
Sex (%)			0.003
Male	34.6	39.7	
Female	65.4	60.3	
Age (years)	40.2 ± 10.1	38.7 ± 10.9	<0.001
Body mass index	22.9 ± 3.9	22.7 ± 3.8	0.078
Smoking status (%)			0.006
Smoking	33.9	29.2	
No smoking	65.5	69.8	
Qualification (multiple answers allowed; %)			
Certified care worker	84.0	76.9	<0.001
Home care worker	31.1	28.3	0.096
Care manager	13.6	15.9	0.086
No certifications	4.5	7.7	<0.001
Years of experience in total (%)			<0.001
<1 years	3.3	1.1	
≥1 years, <10 years	45.9	41.9	
≥10 years, <20 years	40.3	47.6	
≥20 years	10.1	9.4	
Work time (%)			0.724
Full‐time	93.0	92.6	
Part‐time	6.7	7.1	
Work shift system (%)			0.019
Day shift	22.7	27.3	
Two shifts	21.3	21.4	
Three shifts	37.1	33.4	
Irregular three shifts and so on	15.6	14.7	
Total weekly working hours (%)			<0.001
<35 h	4.7	7.3	
≥35 h, <40 h	27.3	32.1	
≥40 h, <45 h	42.8	40.3	
≥45 h	22.6	17.7	
Job stressors			
Job demand (3–12 points)	9.9 ± 1.7	9.3 ± 1.8	<0.001
Job control (3–12 points)	8.1 ± 1.9	7.5 ± 1.9	<0.001
Worksite social support (6–24 points)	15.2 ± 3.5	14.1 ± 3.7	<0.001

^a^ *p*‐value.

### 3.3 Care workers' QWL

QWL was significantly lower in the severe LBP group than in the nonsevere group (11.5 ± 3.0 vs. 12.7 ± 2.7, *p* < 0.001).

### 3.4 Factors of QWL

Table 2 summarizes the associations of QWL level with demographics, job characteristics, and job satisfaction ratings for severe LBP and nonsevere LBP groups as revealed by multiple logistic regression models. In the severe LBP group, human relationships with superiors and colleagues had the strongest influence on QWL (OR: 2.92, 95% CI: 1.66–5.13), followed by salary (OR: 2.39, 95% CI: 1.26–4.54), support from the workplace in case of trouble (OR: 1.99, 95% CI: 1.15–3.43), discretionary responsibility level (OR: 1.91, 95% CI: 1.08–3.38), and working hours or time off (OR: 1.85, 95% CI: 1.02–3.36).

**TABLE 2:** Associations of QWL with demographics, job characteristics, and job satisfaction ratings from multiple logistic regression.

	Severe LBP (*n* = 1247)					Nonsevere LBP (*n* = 2009)				
	OR^a^	95% CI^b^			*p* ^c^	OR^a^	95% CI^b^			*p* ^c^
Sex										
Male	1.00					1.00				
Female	0.87	0.52	–	1.47	0.609	0.80	0.59	–	1.08	0.799
Age										
<30 y	1.00					1.00				
≥30 y, <40 y	0.74	0.36	–	1.54	0.416	0.92	0.60	–	1.42	0.698
≥40 y, <50 y	1.14	0.55	–	2.38	0.723	1.77	1.16	–	2.70	0.008
≥50 y	0.48	0.19	–	1.21	0.117	1.91	1.19	–	3.07	0.008
Total weekly working hours										
<35 h	1.00					1.00				
≥35 h, <40 h	1.63	0.42	–	6.36	0.483	1.09	0.63	–	1.88	0.764
≥40 h, <45 h	1.91	0.50	–	7.33	0.346	0.97	0.56	–	1.68	0.915
≥45 h	1.85	0.46	–	7.49	0.389	0.78	0.41	–	1.46	0.433
Job demand	1.02	0.88	–	1.18	0.843	0.99	0.91	–	1.08	0.837
Job control	0.75	0.65	–	0.87	<0.001	0.91	0.83	–	0.99	0.027
Worksite social support	0.98	0.91	–	1.06	0.605	0.94	0.89	–	0.98	0.005
Human relationships with superiors and colleagues										
Dissatisfaction	1.00					1.00				
Satisfaction	2.92	1.66	–	5.13	<0.001	2.33	1.67	–	3.25	<0.001
Salary										
Dissatisfaction	1.00					1.00				
Satisfaction	2.39	1.26	–	4.54	0.008	1.22	0.84	–	1.77	0.290
Support from the workplace in case of trouble										
Dissatisfaction	1.00					1.00				
Satisfaction	1.99	1.15	–	3.43	0.014	1.73	1.24	–	2.40	0.001
Discretionary responsibility level										
Dissatisfaction	1.00					1.00				
Satisfaction	1.91	1.08	–	3.38	0.026	1.59	1.14	–	2.20	0.006
Working hours/time off										
Dissatisfaction	1.00					1.00				
Satisfaction	1.85	1.02	–	3.36	0.044	1.68	1.20	–	2.33	0.002
Caregiving technique										
Dissatisfaction	1.00					1.00				
Satisfaction	1.39	0.80	–	2.43	0.246	1.47	1.06	–	2.05	0.022
Promotion/official position										
Dissatisfaction	1.00					1.00				
Satisfaction	1.31	0.72	–	2.37	0.373	1.53	1.09	–	2.13	0.013
Personal event										
Dissatisfaction	1.00					1.00				
Satisfaction	1.26	0.73	–	2.16	0.403	0.93	0.69	–	1.27	0.653
Education and training										
Dissatisfaction	1.00					1.00				
Satisfaction	0.96	0.55	–	1.68	0.878	1.16	0.84	–	1.59	0.373
Number of workers										
Dissatisfaction	1.00					1.00				
Satisfaction	0.88	0.28	–	2.78	0.823	1.87	1.16	–	3.03	0.010

^a^ Odds ratio.

^b^ 95% confidence interval.

^c^ *p*‐value.

In the nonsevere LBP group, human relationships with superiors and colleagues was the strongest influence on QWL (OR: 2.33, 95% CI: 1.67–3.25), followed by the number of workers (OR: 1.87, 95% CI: 1.16–3.03), support from the workplace in case of trouble (OR: 1.73, 95% CI: 1.24–2.40), working hours or time off (OR: 1.68, 95% CI: 1.20–2.33), discretionary responsibility level (OR: 1.59, 95% CI: 1.14–2.20), promotion or official position (OR: 1.53 95% CI: 1.09–2.13), and caregiving technique (OR: 1.47, 95% CI: 1.06–2.05).

## 4 DISCUSSION

The present study aimed to identify organizational factors influencing QWL among care workers with severe LBP, one of the most common occupational hazards in this group. The QWL of care workers with severe LBP was slightly lower than that of care workers without severe LBP. Both groups regarded human relationships with superiors and colleagues, support from the facility in case of trouble, discretionary responsibility level, and working hours or time off as significant factors influencing QWL, but only care workers with severe LBP reported salary as a significant influence. In contrast, only care workers without severe LBP reported the number of workers, promotion or official position, and caregiving techniques as significant factors influencing QWL.

Among care workers without severe LBP, the QWL indicated a ‘neutral’ position, which was the median score on the QWL short‐form scale^25^ ranging from 4 to 20. This was consistent with previous studies reporting that QWL and overall job satisfaction ratings by the general care worker population are, at best, mixed.^4^, ^8^, ^30^ Further, ratings were even lower, albeit only slightly, in the group with severe LBP. This difference appears to be related to LBP as basic job characteristics did not differ markedly between groups and several factors strongly influencing QWL were reported by both groups.

Many of the QWL common factors were identified in previous studies. Human relationships^4^, ^8^, ^10^, ^12^, ^14^, ^29^ influence instruction and communication by superiors and colleagues essential for caregiving tasks. Support from colleagues including superiors^5^, ^6^, ^7^ is also helpful for solving problems that may arise in daily practice. In addition, discretionary responsibility^11^, ^12^, ^13^ is essential for any human service requiring flexible and resourceful responses. Working hours and time off^14^ are especially important factors for QWL in seniors' care facilities because it may be difficult to change shifts or obtain unscheduled time off due to chronic staff shortages. We suggest that these factors are shared by most care workers because they directly impact the work environment and the capacity to accomplish routine tasks.

For care workers without severe LBP, promotion or official position, caregiving technique, and the number of workers were also reported as strong influences on QWL. Inappropriate promotion can be discouraging,^5^ while appropriate caregiving techniques enhance work performance and increase both work and patient satisfaction.^31^ An insufficient number of workers obviously increases the individual workload and the capacity for cooperation,^12^ leading to greater stress and lower QWL.^5^, ^14^ Among care workers with severe LBP, however, the salary was also reported as an import for QWL (i.e., care workers who were satisfied with their salary reported higher QWL), consistent with previous studies,^4^, ^8^, ^9^, ^10^, ^14^, ^28^ while factors related to effective caregiving, self‐progress (promotion or official position), and the number of workers were reported as less important. Therefore, care workers with severe LBP require an acceptable salary to endure the pain. However, salary increases may still be insufficient to retain staff with severe LBP, so it is critical to develop occupational safety measures to reduce LBP risk. In addition, institutions should strive to improve helping care workers with severe LBP find worthwhile aims such as smooth caregiving and self‐progress like care workers without severe LBP.

This study has several limitations. Only eight care workers per seniors' care facility were sampled, so the results may have been affected by sampling bias. Since the care workers were supportive of an administrator, their QWL may be higher than the average for all staff. Second, certified care workers accounted for approximately 80% of all participants, and the factors influencing QWL may differ between these more specialized workers and uncertified care workers. Third, results obtained using the QWL short‐form scale^25^ may differ from results using other scales, although QWL scale responses are correlated with those of the work engagement scale in Japanese^26^ and the Japanese burnout scale.^27^ Fourth, this study is a cross‐sectional survey with a low response rate to the questionnaire; therefore, causal associations cannot be mentioned. Further studies are required to address this.

## 5 CONCLUSIONS

The QWL of care workers with severe LBP was slightly lower than that of care workers without severe LBP and was more strongly influenced by salary and less by work practices or the potential for self‐progress. These imply that care workers with severe LBP are working for a salary while enduring the pain and do not have found a worth doing. Institutions need to promote measures to prevent or alleviate severe LBP as well as provide a fulfilling work environment conducive to job satisfaction and self‐progress so that all care workers can continue to work for a long time with motivation.

## AUTHOR CONTRIBUTIONS

K.I. designed and conducted the study, collected and analyzed data, and wrote the manuscript; M.S. collected the data and gave conceptual advice; M.T. analyzed data and gave technical support; X.L. gave conceptual advice. All authors read and approved the final manuscript.

## CONFLICT OF INTEREST

The authors declare that they have no conflict of interest.

## DISCLOSURE

*Approval of the research protocol*: The study aims and protocol were approved by the ethics board of the National Institute of Occupational Safety and Health of Japan (registration ID: H3002). *Informed Consent*: Informed consent was obtained through the questionnaire at the time the data were collected. Participants had the option of not responding to any part of the questionnaire at any time and discontinuing the survey at any point. *Registry and the Registration No. of the study/trial*: This study is not registered. *Animal studies*: N/A.

## Data Availability

All data collected in the study are available upon reasonable request to the authors.
